# A Simple and Highly Sensitive Thymine Sensor for Mercury Ion Detection Based on Surface Enhanced Raman Spectroscopy and the Mechanism Study

**DOI:** 10.3390/nano7070192

**Published:** 2017-07-24

**Authors:** Hao Yang, Sui-Bo Ye, Yu Fu, Weihong Zhang, Fangyan Xie, Li Gong, Ping-Ping Fang, Jian Chen, Yexiang Tong

**Affiliations:** Instrumental Analysis and Research Centre, Ministry of Education of the Key Laboratory of Bioinorganic and Synthetic Chemistry, The Key Lab of Low-Carbon Chemistry & Energy Conservation of Guangdong Province, Key Laboratory of Environment and Energy Chemistry of Guangdong School of Chemistry, Sun Yat-Sen University, 135 Xingang West Road, Guangzhou 510275, China; yanghao9@mail2.sysu.edu.cn (H.Y.); yesuibo@mail2.sysu.edu.cn (S.-B.Y.); fuyu23@mail.sysu.edu.cn (Y.F.); zhangwh@mail.sysu.edu.cn (W.Z.); xiefy@mail.sysu.edu.cn (F.X.); gongli2@mail.sysu.edu.cn (L.G.); fangpp3@mail.sysu.edu.cn (P.-P.F.)

**Keywords:** mercury detection, surface enhanced Raman spectroscopy, sensor, thymine, gold nanorod

## Abstract

Mercury ion (Hg^2+^) is recognized as one of the most toxic metal ions for the environment and for human health. Techniques utilized in the detection of Hg^2+^ are an important factor. Herein, a simple thymine was successfully employed as the surface enhanced Raman spectroscopy sensor for Hg^2+^ ion detection. The limit of detection (LOD) of the developed sensor is better than 0.1 nM (0.02 ppb). This sensor can also selectively distinguish Hg^2+^ ions over 7 types of alkali, heavy metal and transition-metal ions. Moreover, the LOD of the sensor can even achieve 1 ppb in practical application in the nature system, which is half the maximum allowable level (10 nM, 2 ppb) stipulated in the US Environmental Protection Agency standard. Further investigation of the thymine-Hg^2+^-thymine coordination mechanism provides a possible means of detecting other metal ions by replacing the metal ion-specific ligands. This work paves the way for the detection of toxic metal ions and environmental problems.

## 1. Introduction

Mercury ion (Hg^2+^) is one of the most toxic water pollutants, and are not only hazardous to the environment, but also harmful for human health [1,2,3]. Excess accumulation of mercury in the human body can cause neurological disorders, bone softening and other medical effects [4,5]. To date, different types of analytical methods have been developed to achieve better sensitivity and selectivity for Hg^2+^ ion detection, such as colorimetric assays [6,7], fluorescence-based assays [8,9] and inductively coupled plasma-atomic emission spectrometry (ICP-AES) [10,11]. However, traditional optical methods have limitations in terms of sensitivity or selectivity, and often require labeling tags or complicated instruments [12,13].

Among the methods developed for Hg^2+^ ion detection, surface-enhanced Raman spectroscopy (SERS) is a promising candidate due to its distinct advantages in terms of high sensitivity, non-invasiveness, non-labeling, and fingerprint-type [14,15,16]. Recently, several SERS methods have been developed for the Hg^2+^ ion detection based on molecular probes, such as DNA [17,18], protein [19,20], oligonucleotides [21,22] and fluorophores [23,24]. However, these molecular probes are very complicated and expensive [25]. Hence, it is desirable to detect Hg^2+^ ions using a simple and inexpensive small molecular probe. Thymine is a lost-cost and commonly available material, since it can be obtained by simple chemical synthesis [26]. Furthermore, the high stability and low Raman background signal make thymine a prospective alternative for Hg^2+^ ion detection.

Here, we present an outstanding sensitivity and selectivity SERS method for the Hg^2+^ ion detection using a thymine sensor instead of DNA. The developed method can clearly detect Hg^2+^ ions at remarkably low concentrations (0.1 nM, 0.02 ppb), and can selectively distinguish Hg^2+^ ions over 7 other alkali, heavy metal- and transition-metal ions. Importantly, the limit of detection (LOD) in the river water can even achieve at 5 nM (1 ppb), which is half the maximum allowable level (10 nM, 2 ppb) in the US Environmental Protection Agency (EPA) standard [27]. Moreover, the basic mechanism of the Thymine SERS sensor was also investigated, which opens up opportunities for detecting other metal ions by replacing the metal ion-specific ligands.

## 2. Results and Discussion

**Figure 1:**
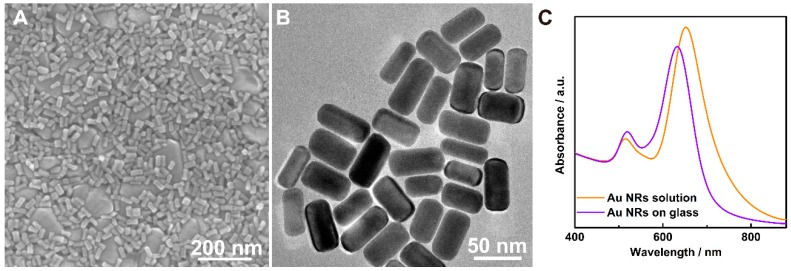
(**A**) SEM image of the Au NRs deposited on glass sheets; (**B**) TEM image of the Au NRs; (**C**) UV-Visible spectra of the Au NRs solution and Au NRs on glass sheet.

The Au nanorods (Au NRs) used in this study were synthesized using the seed-mediated growth method, which has been reported in our previous work [14,28]. Scanning electron microscopy (SEM) images confirm that large-area uniform Au NRs were obtained on the glass sheets for SERS (Figure 1A). The homogeneity of the SERS substrate is an important element in the reproducibility of Raman signals. Further transmission electron microscopy (TEM) studies on the Au NRs samples show that their length is about 50 ± 5 nm (Figure 1B). In addition, the strong and sharp local surface plasmonic resonance peaks were observed in both Au NRs solution and Au NRs on glass sheets in the extinction spectra (Figure 1C), indicating the low level of shape impurities, such as nanospheres and nanoplates.

**Figure 2:**
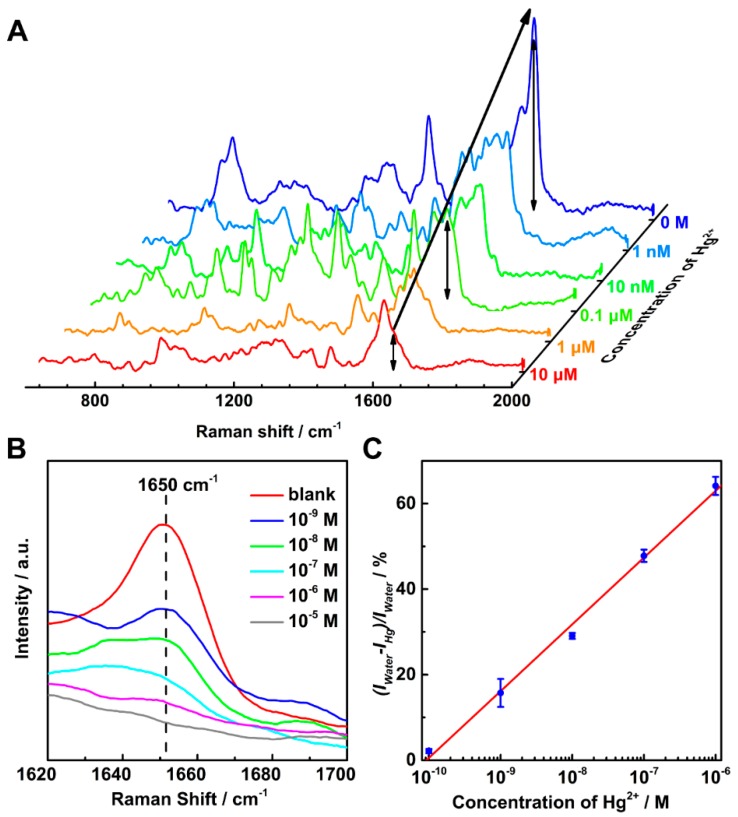
(**A**) SERS spectra of Au NRs@T with different concentrations Hg^2+^ ion; (**B**) The characteristic SERS peak at 1650 cm^−1^ with different concentrations Hg^2+^ ion; (**C**) Variation of SERS intensity at 1650 cm^−1^ as a function of Hg^2+^ ion concentration.

The as-prepared Au NRs deposited on the glass sheets can serve as SERS active substrate by adsorbing thymine, which was denoted as Au NRs@T. The sensitivity of Hg^2+^ ion detection of the Au NRs@T was investigated by measuring the Raman spectra with different Hg^2+^ ion concentrations (1 nM to 10 μM). As shown in Figure 2A, the strongest Raman peak at 1650 cm^−1^ was the characteristic peak of thymine, which can be ascribe to the C=O stretching vibration (other vibrational assignment of thymine on Au NRs can be seen in Table 1) [29]. It was obvious that the intensity of the characteristic peak decreased with the increasing Hg^2+^ ion concentration (Figure 2B). Since the strong signal fluctuations could be found according to the well-known Pareto-like statistics of SERS signals in the single-molecule level [30,31,32], we have done more repeated experiments to confirm the reproducibility of our SERS measurements. As can be seen in Figures S1 and S2 in the supporting information (SI), the as-prepared sensor has good reproducibility in the range from 1 nM to 10 μM.

For further quantitative analysis, the intensity of the characteristic peak at 1650 cm^−1^ after different Hg^2+^ ion adsorption was designated as *I*_Hg_, while the intensity after water (without Hg^2+^ ion) immersed was designated as *I*_water_. Therefore, the relative intensity drop would be (*I*_water_ − *I*_Hg_)/*I*_water_. Figure 2C plots the relative intensity drop in logarithmic scale vs the concentration of Hg^2+^ ion, which shows a good linear correlation (correlation constant ca. 0.993). Moreover, it is worth noting that the distinguishable relative intensity drop of ca. 2% can be detected even the concentration of Hg^2+^ ion as low as 0.1 nM (0.02 ppb), which is at least 4 times more sensitive than many other techniques (see Tables S1 in SI), for example, ICP-AES (0.45 nM) [33] fluorescent sensors (1 nM) [9] colorimetric assays (10 nM) [6] and UV-Vis (1 nM) [13]. Although some complicated composite substrates can detect the ppm level Hg^2+^, the LOD of our thymine-based SERS sensors is 5 times more sensitive than many other DNA based SERS sensor in the simple Au nanostructure substrates (see Table S2 in the SI), such as Au nanowire (0.5 nM) [34], Au nanoparticles(1 nM) [35], Au nanorods (4 nM) [36] and Au microshell (50 nM) [37]. This result indicates that our Au NRs@T is an excellent and simple SERS sensor for Hg^2+^ ion detection.

**Figure 3:**
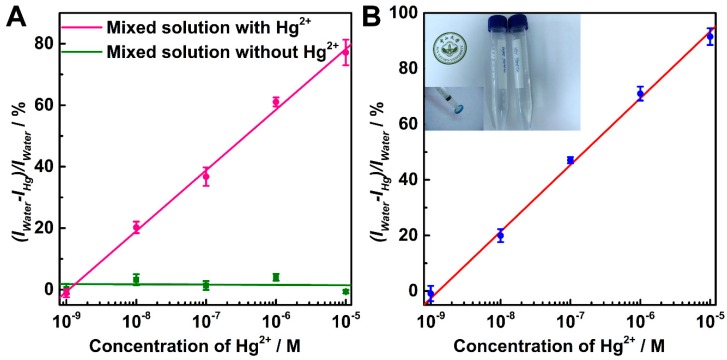
(**A**) Variation of SERS intensity at 1650 cm^−1^ as a function of mental ion concentration. The pink line indicates the solution contain Hg^2+^ and mixed solution, and the green line indicates the solution only contains mixed solution. The mixed solution contains Na^+^, K^+^, Cd^2+^, Zn^2+^, Co^2+^, Cu^2+^ and Fe^3+^. (**B**) Variation of SERS intensity at 1650 cm^−1^ as a function of Hg^2+^ ion concentration in river water. Inset is the river water harvested from Pearl River.

For further investigation on the selectivity of the Au NRs@T, 7 more metal ions were added to the solution, including Na^+^, K^+^, Cd^2+^, Zn^2+^, Co^2+^, Cu^2+^ and Fe^3+^ at the same concentration with Hg^2+^. As depicted in the Figure 3A, there was very little SERS relative intensity drop was observed in the mixed solution without Hg^2+^ ion (3.2% at the concentration of 10 nM), compared to the significant and linear relative intensity drop of the solution with the present of the Hg^2+^ ion (20.3% at the concentration of 10 nM). This result confirms the excellent selectivity of the Au NRs@T with regard to alkali, heavy metal- and transition-metal ions.

The applicability of the Au NRs@T substrate to the nature environments was investigated in the untreated river samples harvested from Pearl River. Hg^2+^ ion was added to the river water to serve as the standard solutions with different Hg^2+^ ion concentration. It should be noted that the Au NRs@T substrate can easily detect 10 nM Hg^2+^ ion with 17.9% of SERS relative intensity drop and also retain the good linear correlation (correlation constant ca. 0.994 at the range from 1 nM to 10 μM) in the river water (Figure 3B). The LOD of the method can achieve 5 nM, which is 2 times lower than the EPA standard [27]. These results indicate that the high sensitivity of the Au NRs@T substrate can even retain in the nature system for practical application.

**Figure 4:**
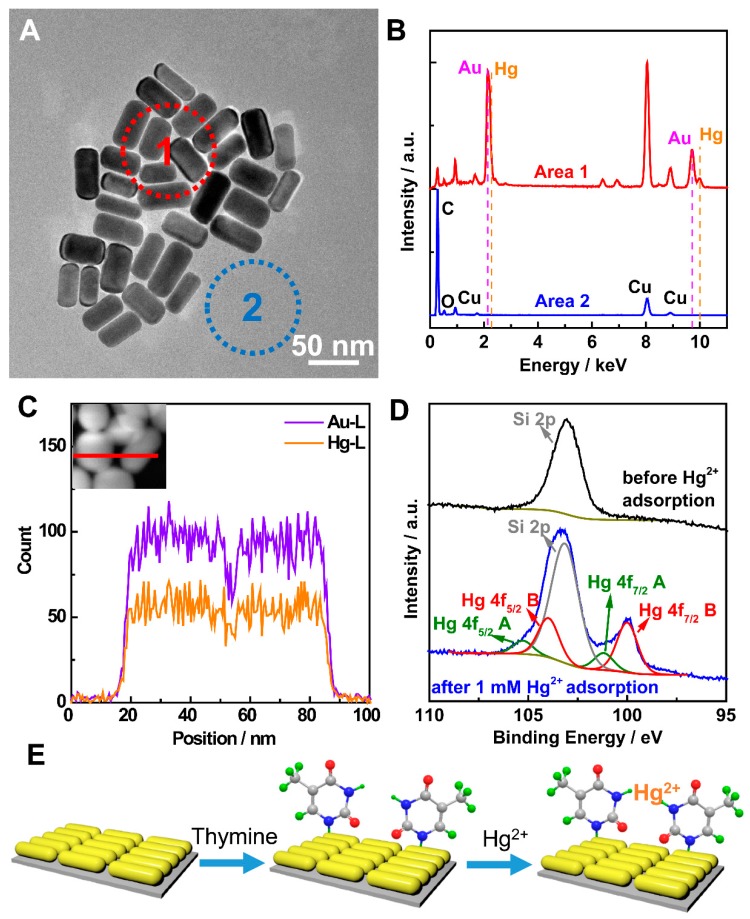
(**A**) TEM image of the Hg^2+^ ion adsorbed on the Au NRs@T and (**B**) corresponding EDS patterns in selected area. (**C**) EDS line scans along with the red line in the inset high-angle annular dark-field scanning transmission electron microscopy image. (**D**) High-resolution XPS spectra of the Au NRs@T before and after Hg^2+^ ion adsorption. (**E**) Schematic illustration of the SERS detection of Hg^2+^ ion on the Au NRs@T.

It is well-known in the reported literature that Hg^2+^ ions can coordinate with thymine. However, most of the reported detection method based on this did not investigated the experimental mechanism [9,17,18]. To further understand the nature of the high sensitivity and selectivity of the SERS method by the as-prepared Au NRs@T substrate, energy dispersive X-ray spectroscopy (EDS) (FEI Co., Ltd., Hillsboro, OR, USA) and X-ray photoelectron spectroscopy (XPS) (Thermo-VG Scientific Co., Ltd., Waltham, MA, USA) were conducted for the sensor after adsorption. The TEM image of Au NRs@T after adsorption is shown in Figure 4A. The corresponding EDS pattern collected from the selected area (red- and blue-dash line circles in Figure 4A) is depicted in Figure 4B. It is obvious that the Hg can only be detected at the area of the Au NRs@T (area 1 in Figure 4A). Furthermore, the EDS line scans in Figure 4C show that the intensity of the Hg is in agreement with the Au, which suggests that the Hg^2+^ ion can be adsorbed at the Au NRs@T.

This adsorption was further investigated by the XPS of the Au NRs@T before and after Hg^2+^ ion adsorption. XPS survey spectra confirmed the presence of Hg after Hg^2+^ ion adsorption (Figure S3). High-resolution XPS spectra of Hg are depicted in Figure 4D. A peak at 100.0 eV in the Hg 4f spectra can be found after Hg^2+^ ion adsorption, which is totally absent before adsorption [38]. Moreover, we observed that there are two type Hg 4f_5/2_ peak and Hg 4f_7/2_ peak group. Group A (green line in Figure 4D), which includes the Hg 4f_5/2_ peak (105.4 eV) and Hg 4f_7/2_ peak (101.2 eV), refers to the Hg^2+^ ion physically adsorbed at the surface of Au NRs@T [38]. It is worth noting that the other type of Hg 4f_5/2_ peak and Hg 4f_7/2_ (Group B, red line in Figure 4D), were shifted to the 104.0 and 100.0 eV, respectively. We attribute these shifts to the fact that the lone pair electrons transfers to the Hg^2+^ ion from thymine, implying that the Hg^2+^ ion was chemically adsorbed on the surface of Au NRs@T. In order to confirm this, we measured the mass spectrum, the results of which are shown in Figure S4. Thymine-Hg^2+^-thymine (T-Hg^2+^-T) base pair at 452 *m*/*z* was recorded, indicating T-Hg^2+^-T coordination. We concluded that the high sensitivity and selectivity of the described SERS method can be attributed to coordination mechanism in Figure 4E. Firstly, thymine was adsorbed at the surface of Au NRs. Secondly, the Hg^2+^ ion can selectively coordinate with thymine to form the stable T-Hg^2+^-T coordination compound. This can be accounted for the high selectivity ion Hg^2+^ ion.

## 3. Materials and Methods

### 3.1. Materials

All chemicals were purchased and used without any further purification. To perform mercury ion quantification assay, mercury nitrate (99%, Guangzhou chemical reagent factory, Guangzhou, China) was dissolved in purified water to prepare aqueous solution with various concentrations. Thymine (Sigma-Aldrich Co., LLC., St. Louis, MO, USA) was dissolved in purified water to prepare solutions of 10^−3^ M. Cetyltrimethyl-ammonium bromide (CTAB, 99%), cadmium chloride (CdCl_2_, 99%) obtained from Kermel (Tianjin, China), toluene (C_7_H_8_, A.R.), hydrochloric acid (37% HCl, A.R.), Ethanol (C_2_H_5_OH, A.R.), potassium nitrate (KNO_3_, 99%), zinc nitrate hexahydrate (Zn(NO_3_)_2_·6H_2_O, 99%), cobaltous nitrate gexahydrate (Co(NO_3_)_2_·6H_2_O, 99%), copper nitrate hydrate (Cu(NO_3_)_2_·3H_2_O, 99%) and iron nitrate nonahydrate (Fe(NO_3_)_3_·9H_2_O, 99%) obtained from Guangzhou chemical reagent factory (Guangzhou, China). Hydrogen tetrachloroaurate (HAuCl_4_ 3H_2_O, 99.99%), sodium borohydride (NaBH_4_, 99.99%), silver nitrate (AgNO_3_, 99.99%) and ascorbic acid (AA, 98%) were purchased from Sigma-Aldrich (Sigma-Aldrich Co., LLC., St. Louis, MO, USA). Sodium nitrate (NaNO_3_, 99%) was purchased from Damao chemical reagent company (Tianjin, China). Millipore water was used through all the experiments.

### 3.2. Preparation of Au NRs

Au NRs were synthesized by seed-mediated growth method, which has been reported in our previous work [14,28]. The ice-cold NaBH_4_ aqueous solution (600 µL, 0.1 M) was rapidly prepared and added into the HAuCl_4_ (250 µL, 10 mM) and CTAB (9.75 mL, 0.1 M) aqueous mixture. Adequate inversion mixing was conducted in each step. At room temperature CTAB-stabilized Au nanocrystal seeds were formed in 3 h. To obtain Au NRs, 10 µL of the as-prepared seed solution was added to 10.67 mL growth solution, which contained HAuCl_4_ (0.47 mM), AgNO_3_ (0.047 mM), HCl (18.74 mM), AA (0.75 mM) and CTAB solution (93.70 mM). The mixture was kept at room temperature for 12 h to grow Au NRs. At the presence of Ag^+^, the aspect ratio of NRs can be tuned by the ratio of gold seed to gold salt.

### 3.3. Fabrication of Thymine Modified Au NRs@T

To modify thymine on the Au NRs, first 4 mL as-prepared Au NRs solution was centrifuged and the supernatant was removed. Then 4 mL toluene was added and kept at 65 °C for 5 min to remove the residual CTAB. 4 mL purified water was added to the mixture after the second centrifugation. After the third centrifugation 4 mL thymine was added to the mixture and 4 glass sheets (1 cm × 1 cm) were immersed detached in the solution. The mixture was allowed to stand overnight for the precipitation of the Au NRs@T. The as-prepared Au NRs@T was used for Hg^2+^ ion detection.

### 3.4. Characterization

The morphology of the probe was measured using Quanta 400/INCA/HKL (FEI Co., Ltd. , Hillsboro, OR, USA). The morphology and EDS line scan was measured by Tecnai^TM^ G2 F30 (TEM) (FEI Co., Ltd., Hillsboro, OR, USA), operating at 300 kV. The area EDS was measured by Tecnai^TM^ G2 Spirit (TEM) (FEI Co., Ltd., Hillsboro, OR, USA), operating at 120 kV. For measuring the extinction spectra of colloidal samples, a SHIMADZU UV-Visible-near infrared spectrophotometer (Shimadzu Corp., Kyoto, Japan) with an incidence spot size of 5 mm was utilized. X-ray photoelectron spectroscopy (Thermo-VG Scientific Co., Ltd., Waltham, MA, USA) was performed to identify the chemical composition of the surface of the observed nanocomposites. The mass spectrometer measurements were carried out using a TSQ Quantum Ultra (Thermo-VG Scientific Co., Ltd., Waltham, MA, USA).

### 3.5. Detection of Hg^2+^ Ion

SERS spectra were measured by a Renishaw inVia (Renishaw plc, New Mills, UK) with a He-Ne laser at 632.8 nm. Each Raman spectrum was obtained using 5% laser power, one accumulation and the acquisition time was typically 10 s. To detect Hg^2+^ ion, 1 M mercuric nitrate solution of was diluted to 10^−1^–10^−10^ M. Secondly, the probes were marked on the surface and SERS spectra of 3–5 points were recorded (The error bar in all figures is equal to the standard deviation of relative intensity drop of the 3–5 points.). Then, the probes were immersed in 60 µL of 0.1 nM to 100 μM of mercuric nitrate solution, respectively. After 5 min standing, the probes were purged with N_2_ and SERS spectra of the same points above were recorded. The background correction was processed by LabSpec (HORIBA, Ltd., Kyoto, Japan). To correspond with the actual application situation, every probe was used once and corresponded to one certain concentration.

## 4. Conclusions

In conclusion, we have developed a simple Au NRs@T SERS method for Hg^2+^ ion detection with high sensitivity and excellent selectivity. In the developed method, thymine was employed as the sensor instead of DNA. Our experimental study demonstrates that the LOD of the developed method is 0.1 nM (0.02 ppb), which is 4 times more sensitive than many other techniques. This method can also selectively distinguish Hg^2+^ ion over 7 types of alkali, heavy metal- and transition-metal ions. Moreover, the LOD of the Au NRs@T SERS method can even achieve 5 nM (1 ppb) in natural environments for practical application, which is half the EPA standard. This makes Au NRs@T substrates ideal candidates for Hg^2+^ ion sensors. Secondly, the further investigation of the T-Hg^2+^-T coordination mechanism provides a possible way to detect other metal ions by replacing the metal ions-specific ligands. This work paves pathway for the detection of toxic metal ions and environmental problems.

## Figures and Tables

**Table 1:** Assignment of SERS spectra for thymine on Au NRs.

Raman Shift (cm^−1^)	Assignment
1650	C=O stretching vibration
1435	N–H deformation
1369	N–H and C–H in-plane bending
1230	Ring stretch
1013	Ring stretch
984	N–H wagging
804	Ring deformation bend
738	Ring breathing

## Supplementary Materials

The following are available online at http://www.mdpi.com/2079-4991/7/7/192/s1, Figure S1: SERS spectra of three Au NRs@T substrates with different concentrations Hg^2+^ ion. (A) 0 M, (B) 0.1 nM, (C) 1 nM, (D) 10 nM, (E) 100 nM and (F) 1 μM. Figure S2: Variation of SERS intensity of three random point on a Au NRs@T substrate as a function of Hg^2+^ ion concentration. Table S1: The LOD of different method for Hg^2+^ ion detection. Table S2: The LOD of SERS methods for Hg^2+^ ion detection. Figure S3: XPS survey of the Au NRs@T before and after 1 mM Hg^2+^ ion adsorption. Figure S4: Mass spectrum of the Au NRs@T after 1 mM Hg^2+^ ion adsorption.
