# Supplementary material for: A Simple and Highly Sensitive Thymine Sensor for Mercury Ion Detection Based on Surface Enhanced Raman Spectroscopy and the Mechanism Study
Source: Nanomaterials (Basel). 2017 Jul 24;7(7):192. doi: 10.3390/nano7070192 (PMC5535258; doi:10.3390/nano7070192)
Supplement: Supplementary file 1 [file nanomaterials-07-00192-s001.pdf]

Supporting information

A Simple and Highly Sensitive Thymine Sensor for Mercury Ion Detection Based on Surface Enhanced Raman Spectroscopy and the Mechanism Study

Hao Yang[1], Sui-Bo Ye[1], Yu Fu[1], Weihong Zhang[1], Fangyan Xie[1], Li Gong[1], Ping-Ping Fang[1], Jian Chen[1,*], and Yexiang Tong[1,*]

Instrumental Analysis and Research Centre, Ministry of Education of the Key Laboratory of Bioinorganic and Synthetic Chemistry, The Key Lab of Low-carbon Chemistry & Energy Conservation of Guangdong Province, Key Laboratory of Environment and Energy Chemistry of Guangdong School of Chemistry, Sun Yat-Sen University, 135 Xingang West Road, Guangzhou 510275, China; yanghao9@mail2.sysu.edu.cn (H.Y.); yesuibo@mail2.sysu.edu.cn (S.-B.Y.); fuyu23@mail.sysu.edu.cn (Y.F.); zhangwh@mail.sysu.edu.cn (W.Z.); xiefy@mail.sysu.edu.cn (F.X.); gongli2@mail.sysu.edu.cn (L.G.); fangpp3@mail.sysu.edu.cn (P.-P.F.)

* Correspondence: puscj@mail.sysu.edu.cn (J.C.); chedhx@mail.sysu.edu.cn (Y.T.); Tel.: +86-020-8411-0788 (J.C.); +86-020-8411-0071 (Y.T.)

Supplementary Caption Lists

Figure S1. SERS spectra of three Au NRs@T substrates with different concentrations Hg^{2+} ion. (A) 0 M, (B) 0.1 nM, (C) 1 nM, (D) 10 nM, (E) 100 nM and (F) 1 μM.

Figure S2. Variation of SERS intensity of three random point on a Au NRs@T substrate as a function of Hg^{2+} ion concentration.

Table S1. The LOD of different method for Hg^{2+} ion detection.

Table S2. The LOD of SERS methods for Hg^{2+} ion detection.

Figure S3. XPS survey of the Au NRs@T before and after 1 mM Hg^{2+} ion adsorption.

Figure S4. Mass spectrum of the Au NRs@T after 1 mM Hg^{2+} ion adsorption.

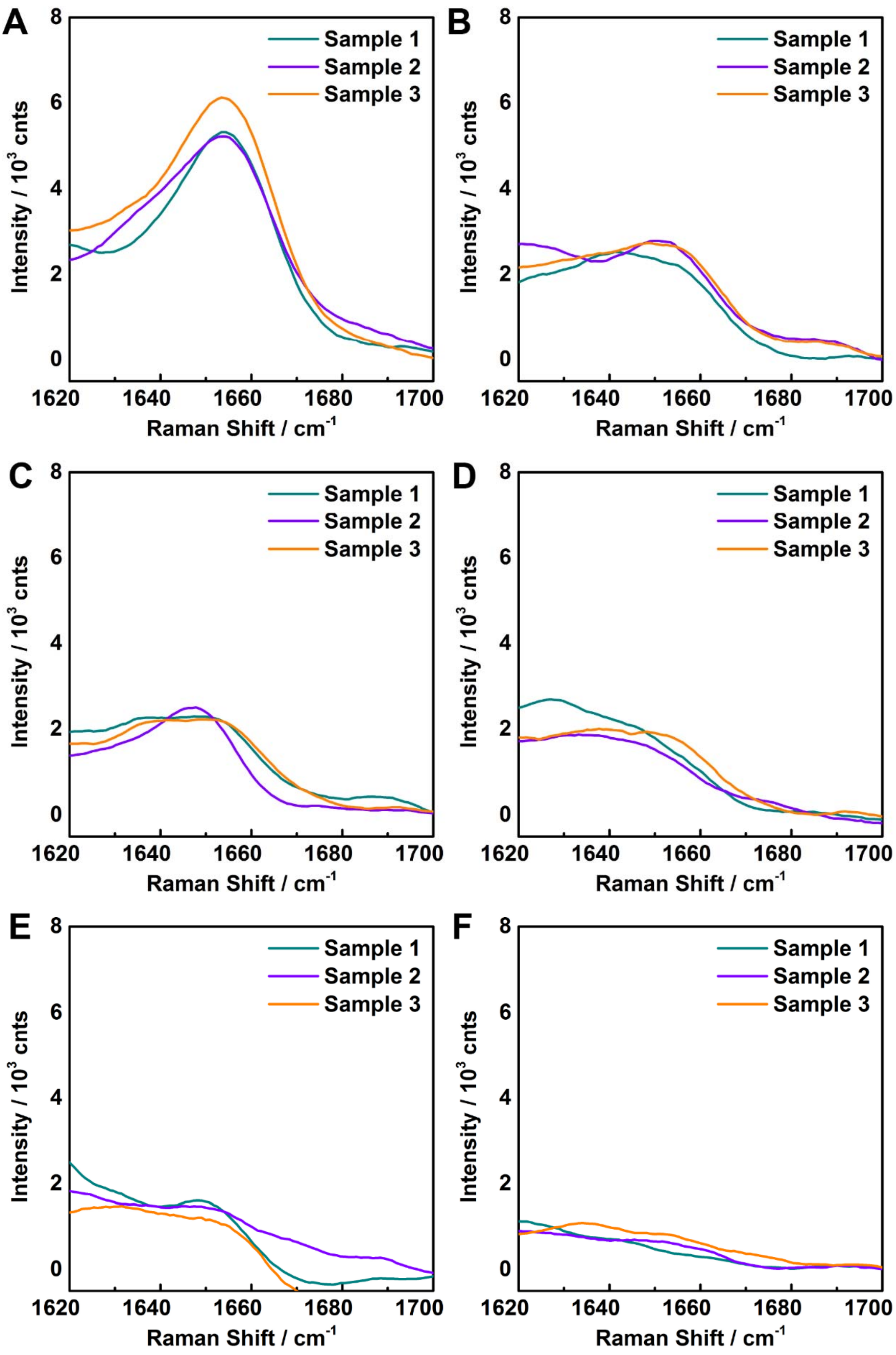

Figure S1. SERS spectra of three Au NRs@T substrates with different concentrations Hg^{2+} ion. (A) 0 M, (B) 0.1 nM, (C) 1 nM, (D) 10 nM, (E) 100 nM and (F) 1 μM.

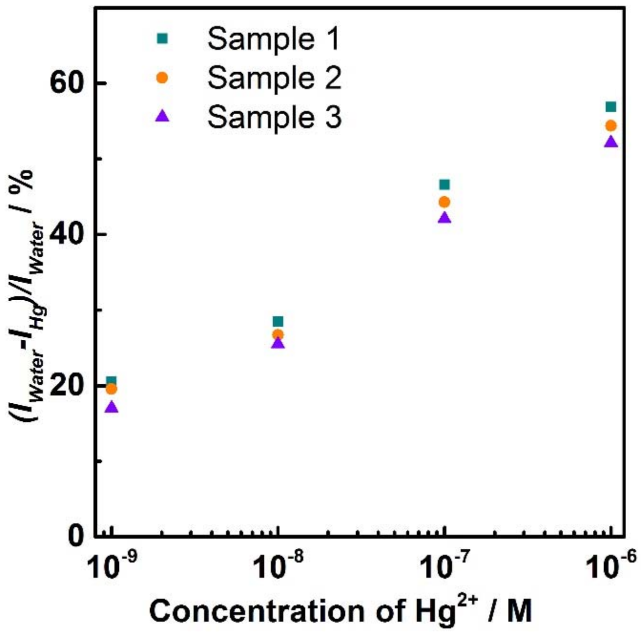

Figure S2. Variation of SERS intensity of three random point on a Au NRs@T substrate as a function of Hg^{2+} ion concentration.

Table S1. The LOD of different method for Hg^{2+} ion detection

Method	LOD	Reference
Ultraviolet visible light absorption spectrometry (UV-Vis)	1 nM (0.2 ppb)	*Angew. Chem. Int. Ed.* **2008**, *47*, 3927.
Inductively Coupled Plasma-Atomic Emission Spectrometry (ICP-AES)	0.45 nM (0.09 ppb)	*Int. J. Environ. Anal. Chem.* **2011**, *91*, 1024.
Metal NPs based fluorescent with DNA sensors	1 nM (0.2 ppb)	*Angew. Chem. Int. Ed.* **2008**, *47*, 8386.
Ag NPs-based colorimetric assays with DNA sensor	10 nM (2 ppb)	*Talanta* **2012**, *97*, 388.

Table S2. The LOD of SERS methods for Hg^{2+} ion detection.

Substrate	LOD	Reference
Au nanorods with thymine sensor	0.1 nM (0.02 ppb)	This work
Au nanoparticles/graphene with DNA sensor	0.1 nM (0.02 ppb)	*ACS Appl. Mater. Inter.* **2013**, *5*, 7072.
Oligonucleotide-functionalized magnetic silica sphere@Au nanoparticles with DNA sensor	0.1 nM (0.02 ppb)	*ACS Appl. Mater. Inter.* **2014**, *6*, 7371.
Ag with DNA and PATP hybrid sensor	0.1 pM (0.02 ppt)	*Chem. Commun.* **2011**, *47*, 9360.
Au nanowire with DNA sensor	0.5 nM (0.1 ppb)	*Lab Chip.* **2012**, *12*, 3077.
Au nanoparticles with DNA sensor	1 nM (0.2 ppb)	*Environ. Sci. Technol.* ***2009**, 43, 5022.*
Au nanoparticles decorated silicon nanowire array with DNA sensor	1 pM (0.2 ppt)	*Anal. Chem.* **2015**, *87*, 1250.
Au nanorods with DNA sensor	4 nM (0.8 ppb)	*Anal. Met.* **2015**, *7*, 4514.
Au@Ag nanoparticles with DNA sensor	5 pM (1 ppt)	*Biosens. Bioelectron.* **2015**, *69*, 142.
Au TNAs/n-Layer graphene/Au nanoparticles sandwich structure with DNA sensor	8.3 nM (1.66 ppb)	*Small* **2017**, *13*. DOI: 10.1002/smll.201603347
Au/Ag core–shell nanoparticles with DNA sensor	10 pM (2 ppt)	*Lab Chip.* **2013**, *13*, 260.
Au microshell with DNA sensor	50 nM (10 ppb)	*Chem. Commun.* ***2010**, 46, 5587.*

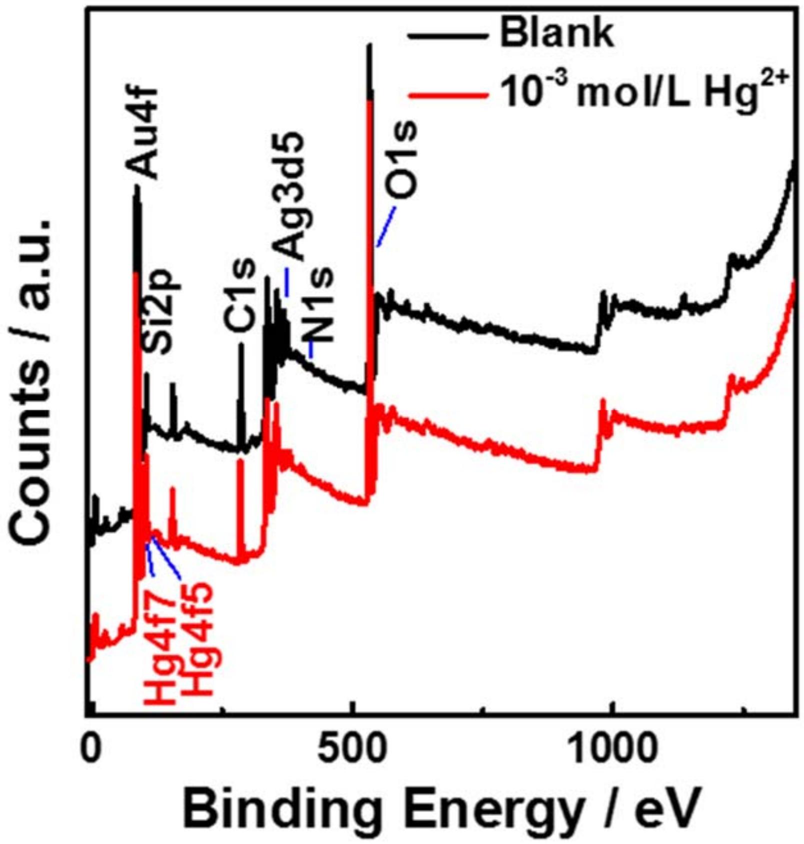

Figure S3. XPS survey of the Au NRs@T before and after 1 mM Hg^{2+} ion adsorption.

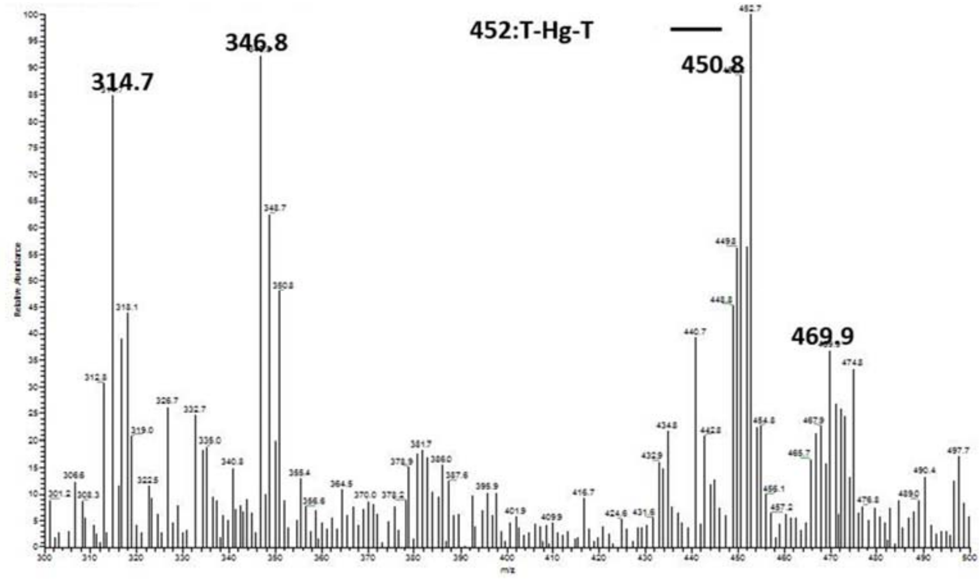

Figure S4. Mass spectrum of the Au NRs@T after 1 mM Hg^{2+} ion adsorption.
